# Supplementary material for: HIVprotI: an integrated web based platform for prediction and design of HIV proteins inhibitors
Source: J Cheminform. 2018 Mar 9;10:12. doi: 10.1186/s13321-018-0266-y (PMC5845081; doi:10.1186/s13321-018-0266-y)
Supplement: Supplementary file 2 — Additional file 2. Source code of HIVProtI web server. [file 13321_2018_266_MOESM2_ESM.zip › HIVprotI_Source-code/hivprotI/weka-3-6-6/doc/weka/classifiers/MultipleClassifiersCombiner.html]

MultipleClassifiersCombiner


---


|  |  |  |
| --- | --- | --- |
| **Overview** \| **Package** \| **Class** \| **Tree** \| **Deprecated** \| **Index** \| **Help** \| **Weka's home** | |  |
| **PREV CLASS**   **NEXT CLASS** | **FRAMES**    **NO FRAMES**     **All Classes** |  |
| SUMMARY: NESTED \| FIELD \| CONSTR \| METHOD | DETAIL: FIELD \| CONSTR \| METHOD |  |


---


## weka.classifiers Class MultipleClassifiersCombiner

```
java.lang.Object
  weka.classifiers.Classifier
      weka.classifiers.MultipleClassifiersCombiner
```

**All Implemented Interfaces:**: java.io.Serializable, java.lang.Cloneable, CapabilitiesHandler, OptionHandler, RevisionHandler

**Direct Known Subclasses:**: RandomizableMultipleClassifiersCombiner

---

``` public abstract class MultipleClassifiersCombiner extends Classifier ```

Abstract utility class for handling settings common to
meta classifiers that build an ensemble from multiple classifiers.

**Version:**
:   $Revision: 5905 $

**Author:**
:   Eibe Frank (eibe@cs.waikato.ac.nz)

**See Also:**: Serialized Form

---

**Constructor Summary**

`MultipleClassifiersCombiner()`


| **Method Summary** | |
| --- | --- |
| `java.lang.String` | `classifiersTipText()`             Returns the tip text for this property |
| `Capabilities` | `getCapabilities()`             Returns combined capabilities of the base classifiers, i.e., the capabilities all of them have in common. |
| `Classifier` | `getClassifier(int index)`             Gets a single classifier from the set of available classifiers. |
| `Classifier[]` | `getClassifiers()`             Gets the list of possible classifers to choose from. |
| `java.lang.String[]` | `getOptions()`             Gets the current settings of the Classifier. |
| `java.util.Enumeration` | `listOptions()`             Returns an enumeration describing the available options |
| `void` | `setClassifiers(Classifier[] classifiers)`             Sets the list of possible classifers to choose from. |
| `void` | `setOptions(java.lang.String[] options)`             Parses a given list of options. |

**Methods inherited from class weka.classifiers.Classifier**

`buildClassifier, classifyInstance, debugTipText, distributionForInstance, forName, getDebug, getRevision, makeCopies, makeCopy, setDebug`

**Methods inherited from class java.lang.Object**

`equals, getClass, hashCode, notify, notifyAll, toString, wait, wait, wait`

**Constructor Detail**

### MultipleClassifiersCombiner

```
public MultipleClassifiersCombiner()
```


**Method Detail**

### listOptions

```
public java.util.Enumeration listOptions()
```

:   Returns an enumeration describing the available options

    :   **Specified by:**: `listOptions` in interface `OptionHandler` **Overrides:**: `listOptions` in class `Classifier`
    :   **Returns:**: an enumeration of all the available options

---


### setOptions

```
public void setOptions(java.lang.String[] options)
                throws java.lang.Exception
```

:   Parses a given list of options. Valid options are:

    -B classifierstring   
    Classifierstring should contain the full class name of a scheme
    included for selection followed by options to the classifier
    (required, option should be used once for each classifier).

    :   **Specified by:**: `setOptions` in interface `OptionHandler` **Overrides:**: `setOptions` in class `Classifier`
    :   **Parameters:**: `options` - the list of options as an array of strings **Throws:**: `java.lang.Exception` - if an option is not supported

---


### getOptions

```
public java.lang.String[] getOptions()
```

:   Gets the current settings of the Classifier.

    :   **Specified by:**: `getOptions` in interface `OptionHandler` **Overrides:**: `getOptions` in class `Classifier`
    :   **Returns:**: an array of strings suitable for passing to setOptions

---


### classifiersTipText

```
public java.lang.String classifiersTipText()
```

:   Returns the tip text for this property

    :   **Returns:**: tip text for this property suitable for displaying in the explorer/experimenter gui

---


### setClassifiers

```
public void setClassifiers(Classifier[] classifiers)
```

:   Sets the list of possible classifers to choose from.

    :   **Parameters:**: `classifiers` - an array of classifiers with all options set.

---


### getClassifiers

```
public Classifier[] getClassifiers()
```

:   Gets the list of possible classifers to choose from.

    :   **Returns:**: the array of Classifiers

---


### getClassifier

```
public Classifier getClassifier(int index)
```

:   Gets a single classifier from the set of available classifiers.

    :   **Parameters:**: `index` - the index of the classifier wanted **Returns:**: the Classifier

---


### getCapabilities

```
public Capabilities getCapabilities()
```

:   Returns combined capabilities of the base classifiers, i.e., the
    capabilities all of them have in common.

    :   **Specified by:**: `getCapabilities` in interface `CapabilitiesHandler` **Overrides:**: `getCapabilities` in class `Classifier`
    :   **Returns:**: the capabilities of the base classifiers **See Also:**: `Capabilities`


---


|  |  |  |
| --- | --- | --- |
| **Overview** \| **Package** \| **Class** \| **Tree** \| **Deprecated** \| **Index** \| **Help** \| **Weka's home** | |  |
| **PREV CLASS**   **NEXT CLASS** | **FRAMES**    **NO FRAMES**     **All Classes** |  |
| SUMMARY: NESTED \| FIELD \| CONSTR \| METHOD | DETAIL: FIELD \| CONSTR \| METHOD |  |


---
